# Simulator Fidelity Does Not Affect Training for Robot-Assisted Minimally Invasive Surgery

**DOI:** 10.3390/jcm12072557

**Published:** 2023-03-28

**Authors:** Shin Saito, Kazuhiro Endo, Yasunaru Sakuma, Naohiro Sata, Alan Kawarai Lefor

**Affiliations:** Department of Surgery, Jichi Medical University, Tochigi 329-0498, Japan

**Keywords:** simulator, fidelity, dry box, surgical robot, GEARS score, virtual reality simulator

## Abstract

This study was undertaken to compare performance using a surgical robot after training with one of three simulators of varying fidelity. Methods: Eight novice operators and eight expert surgeons were randomly assigned to one of three simulators. Each participant performed two exercises using a simulator and then using a surgical robot. The primary outcome of this study is performance assessed by time and GEARS score. Results: Participants were randomly assigned to one of three simulators. Time to perform the suturing exercise (novices vs. experts) was significantly different for all 3 simulators. Using the da Vinci robot, peg transfer showed no significant difference between novices and experts and all participants combined (mean time novice 2.00, expert 2.21, *p* = 0.920). The suture exercise had significant differences in each group and all participants combined (novice 3.54, expert 1.90, *p* = 0.001). ANOVA showed *p*-Values for suturing (novice 0.523, expert 0.123) and peg transfer (novice 0.742, expert 0.131) are not significantly different. GEARS scores were different (*p* < 0.05) for novices and experts. Conclusion: Training with simulators of varying fidelity result in similar performance using the da Vinci robot. A dry box simulator may be as effective as a virtual reality simulator for training. Further studies are needed to validate these results.

## 1. Introduction

The paradigm for surgical education for over 100 years was “see one, do one, teach one”. Despite the fact that surgical training involves learning manual skills, the use of simulation in training was extremely limited until about 2000 when three watershed events occurred almost simultaneously including the institution of work hour restrictions, the Institute of Medicine report and the worldwide clinical application of laparoscopic surgery [1].

In 1993, before the era of robot-assisted surgery, Satava pointed out that surgery simulators must be highly interactive and offer an immersive experience [2]. He predicted the widespread use of head-mounted displays and gloves with electronic sensors. Virtual reality simulators specifically for robot-assisted surgery were reviewed in 2015 by Moglia et al., and the authors lamented the lack of data in this area, particularly to demonstrate skills transfer from simulators to clinical surgery [3].

In a review of virtual reality robot-assisted surgery simulators, Julian et al. reviewed comparative details using available information, of four validated simulators on the market today and specifically described qualitative issues such as visual resolution, available software, scoring systems, price and optional equipment [4]. Face and content validity of the three robot-assisted surgery simulators were evaluated in a single institution study [5]. All three systems had face and content validity with significantly higher scores for the dVSS. The problem with the dVSS is that it depends on access to a da Vinci surgical robot. In a single institution study, surgical fellows compared the dVSS and the Mimic dV Trainer [6]. Results showed significantly higher performance with the dVSS than the dV Trainer. In a review of the current state of virtual reality simulation in robot-assisted surgery, investigators concluded that proficiency-based training is the most effective approach [7].

In a review of 50 studies of robot-assisted surgery, authors concluded that “There is currently no clear advantage with existing robotic platforms, which are costly and increase operative duration. With refinement, competition, and cost reduction, future versions have the potential to improve clinical outcomes without the existing disadvantages.” [8]. In a review comparing open, laparoscopic and robot-assisted pancreas surgery, it was concluded that “Data to show a benefit to the patient are scarce for robotic surgery, although both laparoscopic and robotic surgery of the pancreas have been shown not to be inferior with regard to major operative and oncologic outcomes” [9]. Others have reviewed the results of robot-assisted pancreas surgery and conclude that robot-assisted pancreaticoduodenectomy is the future [10].

Simulator fidelity relates to how well the simulator mimics what is being simulated. There is no single widely accepted definition of fidelity. This concept has been expanded from just concern that high-fidelity simulation requires complete and faithful replication of reality, to the idea that fidelity requires the accurate representation of real-world cues and stimuli [11].

There is a general belief that higher fidelity simulators are “better”, but there is little evidence that higher fidelity simulators provide superior training experiences compared with lower-fidelity simulators as reviewed for laparoscopic simulation [12,13]. This has also been shown for simulation of non-laparoscopic procedures such as colonoscopy [14]. Similar results were seen when comparing results of training with a simple mirrored box to training with a video dry box trainer [15], where training was equal for these two different environments.

It was concluded that simulator fidelity for laparoscopic training does not affect performance after training [12]. In a study of training to perform vascular anastomoses, investigators found that novice operators have greater improvement when using a “high-fidelity” simulator compared to a “low-fidelity” simulator while expert surgeon performance was not dependent on simulator fidelity [16]. There have been no similar studies comparing simulator features for robot-assisted surgery. The use of simulation for teaching surgical skills usually focuses on technical skills. Other forms of training are needed for teaching non-technical skills needed in surgery which demand other forms of simulation. The technical skills needed in surgery can be further divided into basic skills such as those directly related to manual dexterity (e.g., peg transfer exercises) and those skills which are more advanced (e.g., suturing). Simulators of varying fidelity levels have been used for training different types of skills and have been developed to focus on a specific type of training or skill level target.

This study was undertaken to examine the effect of simulator fidelity on training for robot-assisted surgery. Three simulators with various qualitative features are used. The primary outcome of this study is performance on the da Vinci surgical robot after training on a simulator. The secondary outcome of the study is to determine if there is a differential effect on performance for experts compared to novices trained on one of the simulators.

## 2. Materials and Methods

### 2.1. Study Design and Participants

**Figure 1:**
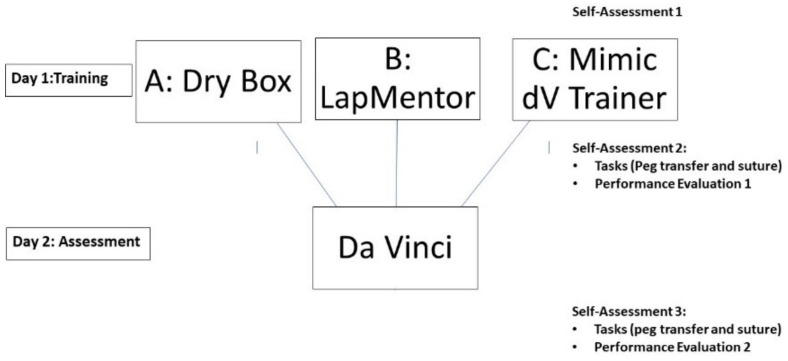
Study Design.

The design of this study is shown in Figure 1. The primary outcome of this study is operator performance on the da Vinci surgical robot. There are three groups of participants, each training with a different simulator. Participants are categorized as novices, who were medical students or first-year residents with no previous surgical experience, and experts, who are senior level residents or board-certified surgeons with surgical experience (general surgery, urology, gynecology). Eight novice operators and 8 experts were randomly assigned to each of the three simulator groups for a total of 48 participants. Participants were randomly assigned to one of three groups corresponding to different simulation training. On the first day, participants practiced on the assigned simulator and then performed two standard tasks including peg transfer and suturing. After the training session, participants returned on a second day to perform the same tasks with the da Vinci robot. This study was approved by the Institutional Review Board of Jichi Medical University before starting.

### 2.2. Simulators

Participants trained using one of three simulators, classified as previously described [12]. Simulator A was a simple simulator, a Type 2 dry box with a light source and video camera connected to a standard definition monitor. A standard grasper and a Maryland dissector were used. Simulator B was a Type 3 virtual reality simulator, the Simbionix LapMentor (3DSystems, Israel https://simbionix.com/simulators/lap-mentor/ accessed on 11 February 2023) which simulates a laparoscopic surgery experience using a computerized image and instruments. The user interface consists of movable wands for which standard surgical instruments can be selected, no kinematic data are recorded and the materials, instruments and image behave in a realistic manner. Simulator C was a Type 3 virtual reality simulator, the Mimic dV Trainer (Mimic Technologies, Seattle WA USA https://mimicsimulation.com/ accessed on 11 February 2023). This simulator produces an image on a video screen that bis similar to the image provided by the da Vinci surgical robot. The user interface is a 3-dimensional movable controller (very similar to the controllers used on the actual da Vinci surgical robot). Standard surgical instruments can be selected by the user, but no kinematic data are recorded. The materials, image and instruments behave in a manner very similar to that experienced when using the da Vinci surgical robot. Thus, these three simulators provide a range of experiences for the user from a simple dry box to a laparoscopic surgical experience to an experience similar to that when using the da Vinci surgical robot.

### 2.3. Exercises

Participants performed the same two exercises, peg-transfer and suturing during the training session (day 1) and in the assessment session (day 2). These exercises were carried out in a manner similar to that described for the Fundamentals of Laparoscopic Surgery program [17]. These 2 exercises were not identical to that conducted in Simulator A because of software limitations on Simulators B and C.

In peg transfer, colored blocks are moved, first from left to right then right to left. All six pegs are moved one way then the other. Each block is picked up with one instrument, transferred mid-air to the other instrument, and deposited on a peg on the opposite side. In the suture exercise, a 3-0 silk suture was pre-cut to 18 cm in length and is then used to suture the two orange rubber tubes together with a single stitch at the top. The suture is placed through the two tubes and then three throws are made to complete the suture. The sutures were not cut. Time for both exercises was measured by a second person observing the exercise. Time intervals from start to finish of each exercise was measured in a similar manner for all participants by the same timekeeper.

These 2 exercises were carried out in a nearly identical fashion on the dry box simulator. Standard laparoscopic graspers and needle holders were used. On Simulator B, the Simbionix LapMentor, the peg transfer exercise looked very similar to the peg transfer block used in the dry box, and the suture was carried out in a similar manner. On Simulator C, the Mimic dV Trainer, the peg transfer was somewhat different with blocks of three colors, and each block was placed in a tray of the same color. There were 12 blocks and each block transferred once to a tray. The suture exercise on Simulator C was carried out in a similar manner as when using the da Vinci robot.

These two exercises are by their nature quite different and based on different skills. The peg-transfer exercise is dependent on manual dexterity and may be considered a basic skill. The suturing exercise is different and depends in part on surgical knowledge and skills acquired in the OR during actual surgery. These differences suggest that the results may differ for novices and experts who have different levels of prior surgical experience.

### 2.4. Assessment

The two exercises were assessed in the same manner on the four platforms used (3 simulators plus the da Vinci robot). As the first assessment, the time to complete each exercise was measured in the same manner by the same timekeeper. As the second assessment, each performance of the two exercises was assessed by two senior surgeons using the GEARS Global Evaluative Assessment of Robotic Skills [18]. The assessments by the two observers are referred to as GEARS1 and GEARS2. As a third assessment, participants completed a self-assessment questionnaire at three time points, prior to the study (Supplementary Table S1), after the training session (Supplementary Table S2) and after the assessment session (Supplementary Table S3). The three questionnaires were translated to Japanese before being administered to participants. The primary endpoint of this study is performance on the da Vinci robot of the two exercises as assessed by time (objective) and the GEARS evaluation (subjective). A secondary endpoint is a comparison of the effects of fidelity for novices compared to experts.

### 2.5. Statistical Analysis

Continuous variables are expressed as means with standard deviation and were compared with the non-parametric Mann–Whitney U-test [19], expressed with *p*-Values. Categorical data (e.g., Likert scale responses on the questionnaire) are expressed as medians with interquartile ranges 1 and 3 (25%ile and 75%ile). Paired data (e.g., novice vs. expert for GEARS scores and times for the two exercises are compared using the Mann–Whitney U-test [19] and results comparing performance on the 3 simulators were compared separately for experts and novices using one-way ANOVA statistics [20].

### 2.6. Sample size

The sample size for this study was determined before it was conducted. Based on α = 0.05 and β = 0.25 (power = 0.80 a minimal sample size of 8 participants per group was calculated. This was based on estimates from [16], which compared results for laparoscopic surgery (there are no comparative studies for robot-assisted surgery) which shows a beta of 0.25 between groups at different levels of training. The power calculation was used to get N for each group. Following the study, a post-hoc power calculation was done as a check on the results using https://clincalc.com/stats/power.aspx (accessed on 11 February 2023). The mean and standard deviation for time in the assessment session (da Vinci robot) with final sample sizes was used as data for this calculator and power calculated.

## 3. Results

### 3.1. Participant Characteristics before the Study

All participants completed a pre-training self-assessment (Supplementary Table S1 and Table 1). The results of this survey show that expert participants were significantly older than novice participants and had more experience after medical school in all 3 training groups. Most of the novice participants were students at the time of the study. Nearly all novice participants had never touched a surgical instrument or tied a knot in a medical context. Most participants were male (40/48). Students judged their surgical ability and confidence as low, while experts judged higher ability and laparoscopic surgery confidence. None of the experts and none of the novices had any previous experience with robot-assisted surgery.

### 3.2. Performance on the Training Exercise

Participants were randomly assigned to one of three simulators (Simulator A, Dry box, Simulator B, LapMentor or Simulator C, Mimic dV trainer) and spent time training on the simulator before a final assessment during the training session. Participants were allowed as much time as they wanted to spend with no fixed curriculum. Participants received assistance one-to-one from a senior surgeon. Performance of the 3 groups on each simulator are shown in Table 2 including GEARS scores and time on the final training exercise.

The groups who trained on simulators A and C had times on the peg transfer that were not significantly different for novices and experts during the training session on day 1. The time to complete the exercises for novices and experts during the suturing exercise were significantly different for groups using all 3 simulators. The GEARS scores for the training exercise were not compared

### 3.3. Participant Characteristics after the Training Exercise

The results of the second self-assessment (Supplementary Table S2) are shown in Table 3, completed after the training exercise. While experts had generally better impressions of their own laparoscopic performance than novices, other questions had good concordance between novice and expert participants. All groups felt that training on the da Vinci would have been preferable although none of them had yet used a da Vinci robot. All groups had relatively low confidence for their performance on the da Vinci robot, but most felt they had done a good job on the training session.

### 3.4. Performance on the Assessment Exercise: Time

The results of time for performing the 2 exercises during the assessment session (da Vinci robot on day 2) are shown in Table 4. The peg transfer exercise had no significant differences between novices and experts for participants in each of the three training groups and no significant difference in the total group (mean time novice 2.13, expert 2.19, *p* = 0.920). The suture exercise had significant differences for time in each of the 3 training groups and for the total group comparing novices and experts (novice 3.54, expert 1.90, *p* = 0.001).

A post-hoc power analysis was performed using the study data with α = 0.05 which showed that the results were significantly different with a power >0.80 for all three training groups as shown in Table 4 (https://clincalc.com/stats/power.aspx accessed on 11 February 2023).

ANOVA analysis was used to compare the times for the three training groups when they used the da Vinci robot. The *p*-Values comparing time for the 3 simulator training groups for novices and experts for suturing (novice 0.523, expert 0.123) and peg transfer exercises (novice 0.742, expert 0.131) were not significantly different, all being >0.05. This shows that there were no significant differences in time performance among the three simulator training groups for performance using the da Vinci robot.

### 3.5. Performance on the Assessment Exercise: GEARS Scores

The results of GEARS scores were determined by 2 independent observers for participants from each training group (GEARS1 and GEARS2), as well as for the total group are shown in Table 4. For each observer there was a significant difference in GEARS scores for novices and experts (*p* = 0.0035 and 0.036).

ANOVA analysis was used to compare the GEARS scores for the three simulator training groups using the da Vinci robot. The *p*-Values comparing GEARS scores for the 3 simulator training groups for novices and experts for GEARS1 (novice 0.486, expert 0.177) and GEARS2 (novice 0.385, expert 0.488) were not significantly different. This shows that there were no significant differences in GEARS scores for participants from each of the three simulator training groups for either of the observers during performance using the da Vinci robot.

### 3.6. Participant Characteristics after the Assessment Exercise

Participants completed the third self-assessment (Supplementary Table S3) after the assessment exercise and results are shown in Table 5. Most groups felt that training would have been better with the da Vinci robot than with the simulator they used. Despite that, most participants were satisfied with the training simulator that they used. Overall, self-assessment of the performance on the da Vinci robot was not high, but the overall confidence level for robot surgery performance was significantly higher for the two groups trained with virtual reality simulators compared to the group trained with a dry box (ANOVA, *p* = 0.012 experts and *p* = 0.0003 novices).

## 4. Discussion

The results of this study show that performance on the da Vinci surgical robot is not related to training on a Class 2, Class 3 with wands or Class 3 robot surgery simulator with a 3-dimensional interface. The study also shows that this lack of an effect is seen for both novice and expert participants, unlike another study which found a differential in training on a “high-fidelity” simulator based on experience [16]. There were 24 novice and 24 expert participants in the present study. Each participant was randomized to use one of the three simulators in the study. The results of the study are reported following randomization of all participants. The absence of a differential in performance based on simulator fidelity was also shown for training in laparoscopic surgery in 8/8 studies that compared 2 levels of simulator fidelity [12] and to the best of our knowledge this is the first study to show a similar result in training for robot-assisted minimally invasive surgery.

One of the problems in simulator comparative studies is the use of relative terms such as “high fidelity” and “low fidelity”. For example, a study compares computer simulation of laparoscopic surgery (classified as “high fidelity”) with a box trainer (“low fidelity”) [13]. A second study compares a video box trainer with a simple box trainer [15]. The low fidelity simulator in the first study is the same as the high fidelity simulator in the second study. This example points out the difficulty with these relative terms. For that reason, a more descriptive system to describe surgery simulators [12] was introduced with a more uniform description of simulators. In that system, all virtual reality simulators are classified together as Type 3 simulators. In the present study, two virtual reality simulators and a dry box trainer were compared, which highlighted the need for an extended classification system for virtual reality simulators. This new classification, referred to as the VERIFY (Virtual Reality Fidelity) indices, is introduced here to serve as a classification scheme for factors that affect the fidelity of virtual reality simulators (Table 6). The factors used in the VERIFY indices were carefully reviewed for four of the available virtual reality simulators [4]. The VERIFY indices relate specifically to use of the instrument and fidelity.

There have been a number of studies thar compare laparoscopic skill performance on simulators with varying fidelity [13,15,21,22,23,24,25,26]. These studies all classify simulators only as “high fidelity” or “low fidelity”. Of the eight studies reviewed, all eight found that performance on laparoscopic skills is not dependent on the fidelity of the simulator used. All eight studies found equal performance in participants who trained on “high-fidelity” and “low fidelity” simulators. This had not been evaluated in training for robot-assisted surgery until the present study, which shows a similar result. This result has significant implications for robot-assisted surgical education and suggests that an inexpensive dry box trainer ca be used equally as effectively for training.

In a review of the effect of simulator fidelity on training for procedures [12], one study showed that junior resident final performance using a checklist assessment on a vascular surgery anastomosis was better for those trained using a “high-fidelity” trainer compared with those trained using a “low fidelity” trainer [16]. It is possible that “low-fidelity” simulators can be used effectively for basic surgical manipulative skill simulation such as peg-transfer but that a “high fidelity” simulator is needed to simulate more advanced skills such as suturing. The present study found no differences in performance on the da Vinci robot by both novice and expert surgeons trained using any of the three simulators tested. This suggests that expert surgeon training can be performed with the same devices used to train novice surgeons. Future studies must be designed to examine the training for different skills with simulators at different fidelity levels.

In a study looking at the correlation between dry lab skills and virtual reality skills, 30 residents completed 5 virtual reality drills on the dVSS and 5 dry lab drills [27]. Dry lab skills were scored with a modified OSATS score. The correlation between virtual reality and dry lab skills showed strong correlation and also had construct validity. These results may partially explain why participants in the present study performed similarly on the virtual reality simulator and the dry box skills. The investigators in [27] suggest that their results support using virtual reality instead of a dry lab, but they do not discuss the cost differential.

In addition to the effects of simulator fidelity on performance, the nature of the exercises performed may also contribute to the observed results. Peg transfer depends on basic skills associated with manual dexterity while suturing is more related to skills learned in surgery and may be more likely to be impacted by prior experience. This difference may partially explain the observed difference in results seen for novices and experts.

Surgical educators in the United States must assure that all residents completing residency training have successfully completed the Fundamentals of Laparoscopic Surgery certification as required by the American Board of Surgery [17]. There has also been an effort to standardize the curriculum for training and assessment of robot-assisted surgeons, highlighted by the Fundamentals of Robotic Surgery program [28]. This effort resulted in a consensus derived set of 25 outcomes measures (pre-operative, intra-operative and post-operative) and a curriculum (cognitive, psychomotor skills and team training) for teaching skills to use robot-assisted surgical systems. The importance of a standardized curriculum as well as a comparison of manual and automated surgeon assessment have been emphasized as well as the need for objective and efficient assessment tools to facilitate training and credentialling of surgeons resulted from an extensive search of the literature [29].

Objective assessment of surgeon performance is an important goal in surgical education, and there have been a number of important contributions in this area. One of the commonly used assessment tools is the Global Evaluative Assessment of Robotic Skills (GEARS) checklist which was developed specifically for robot-assisted surgery [18]. This assessment tool was used in this study to evaluate operator performance in the training and assessment tasks. GEARS is easy to administer and differentiates levels of surgical expertise. This standardized assessment tool shows excellent consistency, reliability and validity [18].

In a comparison of the Mimic dV Trainer and the dVSS, 65 participants completed two trials on a simulator. Investigators found strong correlation between the GEARS score and the simulator metric score for time to complete versus efficiency, time to complete versus total score, economy of motion versus depth perception and overall score versus total score [30]. Investigators concluded that some simulator metrics are well matched with GEARS scores assigned by human reviewers for some virtual reality tasks, and others are not. The importance of objective feedback is emphasized by a study of laparoscopic suturing skills which compared sutures placed by experts and novices [31]. These investigators found a strong correlation between path length and checklist scores, which they conclude would be an objective and comprehensive feedback system.

The sample size for each group in this study was calculated at 8 per group with 6 groups for α = 0.05 and power >0.80 which are typical parameters. A post-hoc power analysis was performed which showed that the power for difference in performance between novices and experts on all 3 simulators is >0.80. It is understood that post-hoc analyses may have limited value, but it also suggests an adequately powered study [32].

While performance on the da Vinci robot after training using the three simulator platforms was not significantly different (Table 4), the results of the self-assessment after the assessment exercise are interesting (Table 3). Specifically, both experts and novice operators scored their confidence level to use the da Vinci robot significantly higher in the two groups which were trained using a virtual reality simulator (Simulators B and C) compared to the group trained using the Dry Box simulator, shown by ANOVA analysis of the responses to the survey. Increased confidence after training with a simulator without improved performance has been shown for other simulation procedural education [33].

Despite the current paucity of data supporting the widespread clinical use of robot-assisted surgery [8], surgical educators and many surgeons concur that this is the future of surgery [10]. A discussion of training is a companion to the above discussion of assessment, since training and assessment are inextricably linked. The Fundamentals of Robotic Surgery curriculum was described above. This curriculum was validated in a single-blinded non-inferiority study which showed better performance of those trained following FRS compared with controls [34]. The authors therefore argue for its implementation across training programs before these skills are used clinically. It will be of interest to see what happens with this curriculum in the future. While the majority of training for laparoscopic surgery in the early 1990s took place in a haphazard fashion, the importance of a defined curriculum for training in robot-assisted surgery has been emphasized [35]. In this extensive literature-based review, authors conclude that validated training curricula, the Global Evaluative Assessment of Robotic Skills and Fundamentals of Robotic Surgery models, have laid the groundwork for a standardized model that can be applied internationally level and across subspecialties. This review provides a foundation from which a future standardized training and credentialing curriculum could be based [35]. Finally, authors conclude that there is a need for a standardized curriculum to be developed and employed for the use of training and credentialing robot-assisted surgeons.

In a study that included 25 novice participants, participants completed peg transfer and suture tasks using a da Vinci surgical robot and then practiced on the dVSS virtual reality simulator, and then performed the tasks using a da Vinci robot [36]. Strengths of this study include the use of a pre-test and post-test as well as use of the dVSS. The authors conclude that novices can attain proficiency using a virtual reality simulator which leads to improved performance in the da Vinci surgical platform on simulated tasks.

The type of training using a virtual reality simulator is also important. The majority of virtual reality trainers for robot-assisted surgery offer training in basic surgical skills such as peg transfer or suturing. A study was performed to compare the effectiveness of structured procedure based virtual reality training with basic virtual reality training and no training [37]. Twenty-six novice participants were randomized to procedure-based or basic skill virtual reality training and then performed part of a urologic procedure on a cadaver. Their performances were compared with 9 participants who had no training. Learning curve analysis demonstrated improved technical skill for both training modalities although procedural training was associated with greater training effects. Any virtual reality training resulted in significantly higher GEARS scores than no training. Procedure-based virtual reality training was found to be more effective than both basic virtual reality training and no training based on GEARS scores. This trial showed that a structured program of procedure-based virtual reality simulation is effective for robot-assisted surgery training with technical skills successfully transferred to a clinical task in cadavers.

The lack of any effect on performance using the da Vinci robot after training on all three simulators was an unexpected result that must be accounted for in the design of future studies. One possible explanation is that the training effect was similar with all three simulators. It is also reasonable to hypothesize that none of the simulators used had any measurable effect. Future studies must include assessment of a training effect, to determine if it exists using the training program offered. This should include assessment of participant performance using the da Vinci robot system before receiving any training. The study also needs a group of equal size where both experts and novices receive no training using a simulator and go directly to the da Vinci robot.

This study has acknowledged limitations. There was no assessment of the training effect during the training session, so the similar performance of the three groups using the da Vinci robot could be due to a complete lack of a training effect. The primary outcome was performance using the da Vinci robot. No participant had previous experience with robot-assisted surgery, which obviated the need for a pre-test since all participants had the same level of experience. It is possible that a difference would have been shown after training with the 3 simulators used if the exercises had been more complex or more limited to robot-assisted surgery instrumentation. There was no specific curriculum or period of time defined for the training session. Future studies should assess the training effect before assessment using the da Vinci robot.

## 5. Conclusions

The results of the present study show that participants trained using three different simulators with varying levels of fidelity all had similar performance when performing standard exercises using the da Vinci surgical robot, suggesting that any of the simulators are effective. Performance among novices and experts was similar no matter which simulator was used, suggesting no differential effect based on simulator fidelity. Taken together these results have important implications for the design and conduct of training programs for robot-assisted surgery. Similar training may be possible with less expensive simulators, although participant confidence was higher after training with a virtual reality system. To obviate confusion regarding simulator classification in the future the previous classification has been extended to include the five VERIFY indices to specifically describe virtual reality simulators (Table 6) including the user interface, instrument function, kinematic data collection, material appearance and anatomic accuracy. Studies comparing simulators should classify simulators with this simple and descriptive system and should no longer use the arbitrary classifications of “high-fidelity” and “low-fidelity”.

## Figures and Tables

**Table 1:** Participant characteristics before training (Self-Assessment 1).

	Dry Box Training		LapMentor Training		Mimic dV Trainer Training	
Characteristic	Novice	Expert	Novice	Expert	Novice	Expert
Male	4/8	8/8	8/8	6/8	7/8	7/8
Age, years	24.9	40.3	23.4	39.8	23	29
Years after medical school	0.5	12.6	0	12.8	0	2.5
Number of open operations	-	381	-	701	-	45
Number of laparoscopic operations	-	80	-	161	-	20
Number of robot-assisted operations	-	0	-	0	-	0
Hours per week of video games	0.56 (3/8)	0.25 (1/8)	1.6 (1/5)	0	6.75 (6/8)	3.8 (3/5)
Survey Questions (1 = low, 7 = high)						
Surgical ability	1 (1, 1)	3 (2, 4)	1 (1, 1.75)	4 (4, 4.25)	4 (2.75, 4)	2 (1.5, 3)
Laparoscopic surgery confidence	1 (1, 1)	2 (2, 3)	1.5 (1, 2)	3.5 (2.5, 4)	4 (2.75, 4.25)	2 (1, 2.5)
Robot-assisted surgery confidence	1 (1, 1)	1 (1, 1.5)	1 (1, 1.75)	1 (1, 1.25)	4 (3, 4.25)	1 (1, 2)

Values shown are mean values for the Likert scale. Likert scale values are median [Interquartile range 1, 3].

**Table 2:** Peg transfer and suture exercises performed using each simulator (day 1).

Training Device	Experience	N	GEARS 1	GEARS 2	Peg Transfer	Suture
Dry box	Novice	8	15.5 ± 2.87	14.4 ± 4.59	2.34 ± 0.62	4.55 ± 1.96
Dry box	Expert	8	22.5 ± 2.87	17.1 ± 3.91	1.80 ± 0.40	2.22 ± 0.93
Novice vs. Expert	*p*-Value				0.17	0.01
LapMentor	Novice	8	17.8 ± 2.65	13.0 ± 2.24	3.31 ± 0.81	6.60 ± 1.52
LapMentor	Expert	8	23.8 ± 1.21	20.5 ± 3.53	2.08 ± 0.34	2.78 ± 1.19
Novice vs. Expert	*p*-Value				0.002	0.002
Mimic dV Trainer	Novice	8	19.4 ± 2.50	14.4 ± 2.51	0.84 ± 0.31	4.34 ± 1.08
Mimic dV Trainer	Expert	8	21.0 ± 2.44	13.6 ± 2.37	0.89 ± 0.72	2.27 ± 0.60
Novice vs. Expert	*p*-Value				0.453	0.0032

Times are shown in decimal minutes; N is number of participants in that group; values are shown as ± standard deviation. GEARS: Global Evaluative Assessment of Robotic Skills, values shown are total scores. GEARS 1: Scores reported by Observer #1; GEARS 2: Scores reported by Observer #2 for that activity.

**Table 3:** Survey after training session (Self-Assessment 2).

	Dry Box Training		LapMentor Training		Mimic dV Trainer Training	
Characteristic	Novice	Expert	Novice	Expert	Novice	Expert
Hours spent on training	2.6	5.7	1.3	5.6	1.21	0.8
Likert Scale Questions (1 = low, 7 = high)						
Laparoscopic confidence after training	1 (1, 2)	3 (2.75, 4)	1 (1, 2.5)	3.5 (2, 4)	3.5 (2.75, 4)	2.0 (2, 3)
Robot-assisted confidence after training	1 (1, 2)	1 (1, 1.3)	1.5 (1, 2.75)	1.5 (1, 2)	3.0 (3, 4)	2.0 (1.5, 2.5)
Training would be better with da Vinci robot	3.5 (1.75, 4)	5 (4, 5.6)	5 (4.25, 5.75)	5 (3.75, 5)	5.5 (5, 6.25)	5.0 (4, 6)
Self-Assessment of training performance	6 (5, 7)	5 (5, 5.8)	6 (5.25, 6)	5 (4, 6.25)	6.0 (5.75, 6.25)	6.0 (6, 7)

Values shown are mean values. Likert scale values are median [Interquartile range 1, 3].

**Table 4:** Peg transfer and suture exercises performed on the da Vinci robot system (day 2).

Training Device	Experience	N	GEARS 1	GEARS 2	Peg Transfer	Suture
Dry box	Novice	8	20.4 ± 2.77	17.1 ± 3.56	1.97 ± 0.43	3.86 ± 1.38
Dry box	Expert	8	24.6 ± 3.66	20.9 ± 5.40	2.78 ± 1.60	2.00 ± 0.40
Novice vs. Expert	*p*-Value (power)				0.12	0.013 (0.956)
LapMentor	Novice	8	20.1 ± 1.64	18.3 ± 2.60	2.20 ± 0.59	3.20 ± 0.90
LapMentor	Expert	8	25.1 ± 3.22	20.1 ± 4.22	1.81 ± 0.23	1.77 ± 0.42
Novice vs. Expert	*p*-Value (power)				0.36	0.0019 (0.983)
Mimic dV Trainer	Novice	8	21.8 ± 3.77	19.75 ± 4.71	2.23 ± 1.02	3.55 ± 1.09
Mimic dV Trainer	Expert	8	22.3 ± 1.38	23.1 ± 5.14	1.94 ± 0.47	2.25 ± 0.47
Novice vs. Expert	*p*-Value (power)				0.818	0.007 (0.872)
All Novice Participants		24	20.9 ± 2.89	18.5 ± 3.76	2.13 ± 0.70	3.54 ± 1.13
All Expert Participants		24	23.9 ± 3.34	21.4 ± 4.97	2.19 ± 1.04	1.90 ± 0.48
Novice vs. Expert	*p*-Value (power)		0.0035	0.036	0.920	0.001 (1.00)

Times are shown in decimal minutes; N is number of participants in that group; values are shown as ± standard deviation. GEARS: Global Evaluative Assessment of Robotic Skills, values shown are total scores. GEARS 1: Scores reported by Observer #1; GEARS 2: Scores reported by Observer #2 for that activity.

**Table 5:** Survey after assessment session (Self-assessment 3).

	Dry Box Training		LapMentor Training		Mimic dV Trainer Training	
Likert Scale Questions (1 = low, 7 = high)	Novice	Expert	Novice	Expert	Novice	Expert
Training would have been better with the da Vinci robot	4.5 (4, 5.25)	5.5 (5, 6)	6.5 (6, 6.75)	5.5 (4.75, 6.25)	6.0 (5.75, 7)	7 (6.25, 7)
I was satisfied with the training device I used	6.5 (5.5, 7)	4.0 (4, 4.5)	5.5 (5, 6)	4.5 (4.75, 6)	5.0 (4.5, 6.25)	6.5 (5.25, 7)
Self-assessment of performance with the da Vinci robot	1.5 (1, 2)	2.5 (1.75, 3)	3 (2, 5)	2.0 (1.75, 2.25)	3.5 (3, 5)	3 (2.25, 3.75)
Confidence to use the da Vinci robot	1.0 (1, 2)	2.0 (1.75, 2.25)	3.5 (2.75, 4.5)	2.0 (1.75, 2)	4.0 (3.75, 5.25)	3.0 (2.25, 4)

Values shown are mean values. Likert scale values are median [Interquartile range 1, 3].

**Table 6:** VERIFY (Virtual Reality Fidelity) indices classification of Type 3 virtual reality robot-assisted surgery simulators.

Index	Liver Simulator	LapMentor	Mimic dV Trainer	ROSS	dVSS
User Interface	X-Box controller and 3D controller and simple joysticks	3D controller, laparoscopic wands	3D controller, similar to da Vinci robot	3D controller, similar to da Vinci robot	3D controller using the da Vinci robot
Instrument Function	2 instruments, ultrasonic shears with realistic function	Multiple, with realistic function	Multiple, with realistic function	Multiple, with realistic function	Multiple, with realistic function
Kinematic Data	YES- standard format, same as da Vinci	YES- proprietary format	YES- proprietary format	YES- proprietary format	YES- proprietary format
Material Behavior	Realistic to touch/cut	Realistic to touch/cut	Realistic to touch/cut	Realistic to touch/cut	Realistic to touch/cut
Anatomic Accuracy	Yes	Yes	Yes	Yes	Yes
Web Site	-	https://simbionix.com (Accessed on 11 February 2023)	https://mimicsimulation.com/ (Accessed on 11 February 2023)	http://simulatedsurgicals.com/projects/ross/ (Accessed on 11 February 2023)	https://mimicsimulation.com/ (Accessed on 11 February 2023)

## Data Availability

All data collected in this study are reported. The original data can be obtained from the corresponding author upon request.

## Supplementary Materials

The following supporting information can be downloaded at: https://www.mdpi.com/article/10.3390/jcm12072557/s1, Additional information are provided in Supplementary Tables.
