# Supplementary material for: Simulator Fidelity Does Not Affect Training for Robot-Assisted Minimally Invasive Surgery
Source: J Clin Med. 2023 Mar 28;12(7):2557. doi: 10.3390/jcm12072557 (PMC10095363; doi:10.3390/jcm12072557)
Supplement: Supplementary file 1 [file jcm-12-02557-s001.zip › jcm-2258607-supplementary.pdf]

**Supplementary Table S1**

# Self-Assessment #1. Robotic Surgery Training Study (Before)

Student Resident Faculty

Number: ______________

M / F

Age: __________________

Year graduate medical school : ___________

Years of Residency completed : __________

Number of open operations: ____________

Number of Laparoscopic Operations: __________

Number of Robotic Operations: ____________

Hours per week of video games (average for last 5 years): ____________

(Before Page 2)

Surgical ability self-assessment:

1-------------------2------------------3-----------------4----------------5-----------------6------------------7

Low Average High

Confidence to perform Laparoscopic Surgery

1-------------------2------------------3-----------------4----------------5-----------------6------------------7

Low Average High

Confidence to perform Robotic surgery

1-------------------2------------------3-----------------4----------------5-----------------6------------------7

Low Average High

**Supplementary Table S2**

## Self-Assessment #2. Robotic Surgery Training Study (After Training on simulator)

Number: ______________

Trainer used : Dry Box Lap Sim Mimic VR DaVinci

Number of Hours on Trainer: ______________

Confidence with laparoscopic surgery

1-------------------2------------------3-----------------4----------------5-----------------6------------------7

Low Average High

Confidence with using the DaVinci after the simulation

1-------------------2------------------3-----------------4----------------5-----------------6------------------7

Low Average High

If you did not train with the DaVinci itself:

"Training with the DaVinci would have been better than the training I used"

1-------------------2------------------3-----------------4----------------5-----------------6------------------7

Disagree No opinion Strongly Agree

Self- Assessment of Training Performance

1-------------------2------------------3-----------------4----------------5-----------------6------------------7

Low Average High

**Supplementary Table S3**

## Self-Assessment #3. Robotic Surgery Training Study (After DaVinci Assessment)

Number: ___________

Trainer used : Dry Box Lap Sim Mimic VR DaVinci

If you did not train with the DaVinci:

"Training with the DaVinci would have been better than the training I used"

1------------------2-----------------3----------------4---------------5----------------6-----------------7

Disagree No opinion Strongly Agree

I was satisfied with the simulation training I received before using the DaVinci

1------------------2-----------------3----------------4---------------5----------------6-----------------7

Disagree No opinion Strongly Agree

Self-Assessment of DaVinci Performance

1------------------2-----------------3----------------4---------------5----------------6-----------------7

Low Average High

Confidence with using the DaVinci

1------------------2-----------------3----------------4---------------5----------------6-----------------7

Low Average High
